# Effects of the frailty phenotype on post-operative complications in older surgical patients: a systematic review and meta-analysis

**DOI:** 10.1186/s12877-019-1153-8

**Published:** 2019-05-24

**Authors:** Binru Han, Qiuping Li, Xi Chen

**Affiliations:** Department of Nursing, Xuanwu Hospital, Capital Medical University, Beijing, China

**Keywords:** Frailty phenotype, Postoperative complications, Geriatric patients, Surgery

## Abstract

**Background:**

Frailty has been generally been associated with adverse events in older patients under surgery. Frailty phenotype is the most widely used instrument in the research literature. However the effect of the frailty phenotype on post-operative events was still unclear. The purpose of this systematic review was to explore the association between frailty phenotype and post-operative complications among surgical patients aged 60 years and over.

**Methods:**

Relevant studies were identified by systematically searching of PubMed, Embase, the Cochrane Library and the Web of Science databases from their beginning to March 2017. Both random-effects models and fixed-effects models were used to combine the risk ratios (RRs) and 95% confidence intervals (CIs). A subgroup analysis was performed to identify the sources of heterogeneity and a sensitivity analysis to identify the strength of the results.

**Results:**

Twelve prospective cohort studies involving a total of 2278 patients were included. The risk of post-operative complications in the frail group was higher than the non-frail group (RR: 1.6; 95% CI: 1.60–2.13). Compared with the robust group, geriatric patients with frailty or pre-frailty had a higher risk of post-operative complications. The RRs were 1.77 (95% CI: 1.40–2.25) and 1.45 (95% CI: 1.17–1.80), respectively.

**Conclusion:**

Frailty phenotype should be considered as a useful risk assessment tool for preoperative evaluations of geriatric patients by medical staff.

## Background

As the size of the older population increases, the rate of surgical procedures in the elderly has been rising. In the USA, about 41% of the people who underwent surgery were over the age of 65 years, and similar statistics are reported from England and some developing countries [1, 2]. However, despite the great progress that has been made in surgical and anesthetic techniques, post-operative morbidity and mortality still remain elevated in the elderly compared to younger patients [3]. It is essential to perform detailed preoperative evaluations to understand the likelihood of surgical morbidity and mortality. Age seems to be related to the risks of surgery, and some researchers have focused on chronological age as an effective assessment tool [4]. However, some recent studies demonstrate that age itself has no influence on adverse post-operative outcomes [5]. It appears that older patients of the same age don’t have similar surgical risks, and elderly people can display significant heterogeneity of risk factors, leading to the emerging hypothesis that “frailty” can be a predictor of adverse post-operative complications in gerontology.

Frailty is defined as a state of reduced reserve and resistance to stressors, resulting from cumulative declines across multiple organ systems, leading to the higher incidence of adverse outcomes [6]. Since 2014, many systematic reviews have been carried out to evaluate the impact of frailty on surgical outcomes in elderly patients. The association between frailty and adverse surgical events has been established [7–9]. There were 20 different frailty assessments have been applied by different research groups [10] However, lacking a universally accepted definition of frailty and mean to measure frailty is problematic. It is difficult for clinicians to choose an effective frailty assessment tool and this may partly explain why frailty has not been used routinely in the preoperative assessment of surgical patients. The frailty phenotype defined by Buta is the most widely used frailty instrument in the research literatures [11]. It includes five components: unintentional weight loss, weakness, exhaustion, slow gait, and low levels of physical activity. Patients who meet 3 or more of these features are deemed frail, while those who have 1 or 2 of the features are deemed “pre-frail” and those without any of the 5 features are called “non-frail” [12]. The frailty phenotype is a quick and convenient scale to ascertain and is thought to be useful in clinical settings. Nevertheless, no meta-analysis has been found in the literature on associations between frailty phenotype and postoperative complications, whether this assessment can accurately predict the incidence of post-operative complications remains elusive.

In order to provide a rational basis for the selection of a frailty assessment tool for surgical risk evaluation in the elderly patients, this meta-analysis was designed to explore the relationship between frailty phenotype and the incidence of post-operative complications among older patients.

## Methods

We conducted a systematic review and meta-analysis of observational studies in accordance with Cochrane Systematic Review guidelines and have reported our findings according to PRISMA reporting guidelines.

### Search strategy

Research publications were obtained from PubMed, Embase, Cochrane Library and Web of Science for the period from the beginnings of the databases until March 2017. Search term combinations were “frail*” AND (“surg*” OR “operat*”) AND (“complication” OR “morbidit*” OR “mortalit*” OR “outcome” OR “death” OR “die*” OR “survival”). In addition, references from relevant articles were reviewed in order to identify potentially useful citations. Only English language, human research, full-length published articles were considered.

### Inclusion and exclusion criteria

The inclusion criteria for this search were (a) original prospective or retrospective cohort studies; (b) individuals aged ≥60 years who underwent operation; (c) frailty was defined by Fried’s frailty phenotype, and divided into two groups (frail and non-frail) or three groups((robust, pre-frail, frail) [12]; and (d) reported post-operative complications based on all definitions. Duplicate articles, review articles, conference abstracts, and letters to the editor were excluded.

### Data collection and analysis

Two researchers (H Br, L Qp) conducted independent searches and evaluated the articles to select eligible studies. The following information was included: author, year of publication, study location, age of participants at the time of inclusion, sample size, percentage of subjects who were male, type of surgery, post-operative complications, and number of events. Furthermore, the same two researchers abstracted the available data, and for any divergence of opinion they consulted a third independent researcher.

### Quality assessment

The Newcastle-Ottawa Scale (NOS) was used to identify the article’s quality. This scale has been validated for the assessment of observational studies [13]. Two researchers (H Br, L Qp) made the quality assessments independently as well. The NOS applied for the cohort study included 3 domains: selection (0–4 points), comparability (0–2 points), and outcome (0–3). The maximum total grade was 9, and a higher grade represented a better study quality.

### Statistical analysis

The strength of the relationship between frailty and post-operative complications was expressed as risk ratios (RRs) with 95% confidence intervals (CIs) in this systematic review and meta-analysis. The statistical heterogeneity across studies was assessed with Cochran’s Q test within using chi-square and I^2^ statistics. For the I^2^ statistics, > 50% indicates moderately heterogeneous results and > 75% is considered highly heterogeneous. When I^2^ was ≤50%, a fixed effect model was used. If not, we analyzed the reason for heterogeneity first and chose a random effects model. Potential publication bias was assessed with funnel plots, Egger’s tests and Begg’s tests. Subgroup analysis was performed to analyze the causes for heterogeneity. All data analyses were conducted with Review Manager version 5.2 (The Nordic Cochrane Centre, Copenhagen, Denmark) and Stata version 12.0 (Stata Corp, College Station, Texas, USA).

## Results

### Study characteristics

**Fig. 1:**
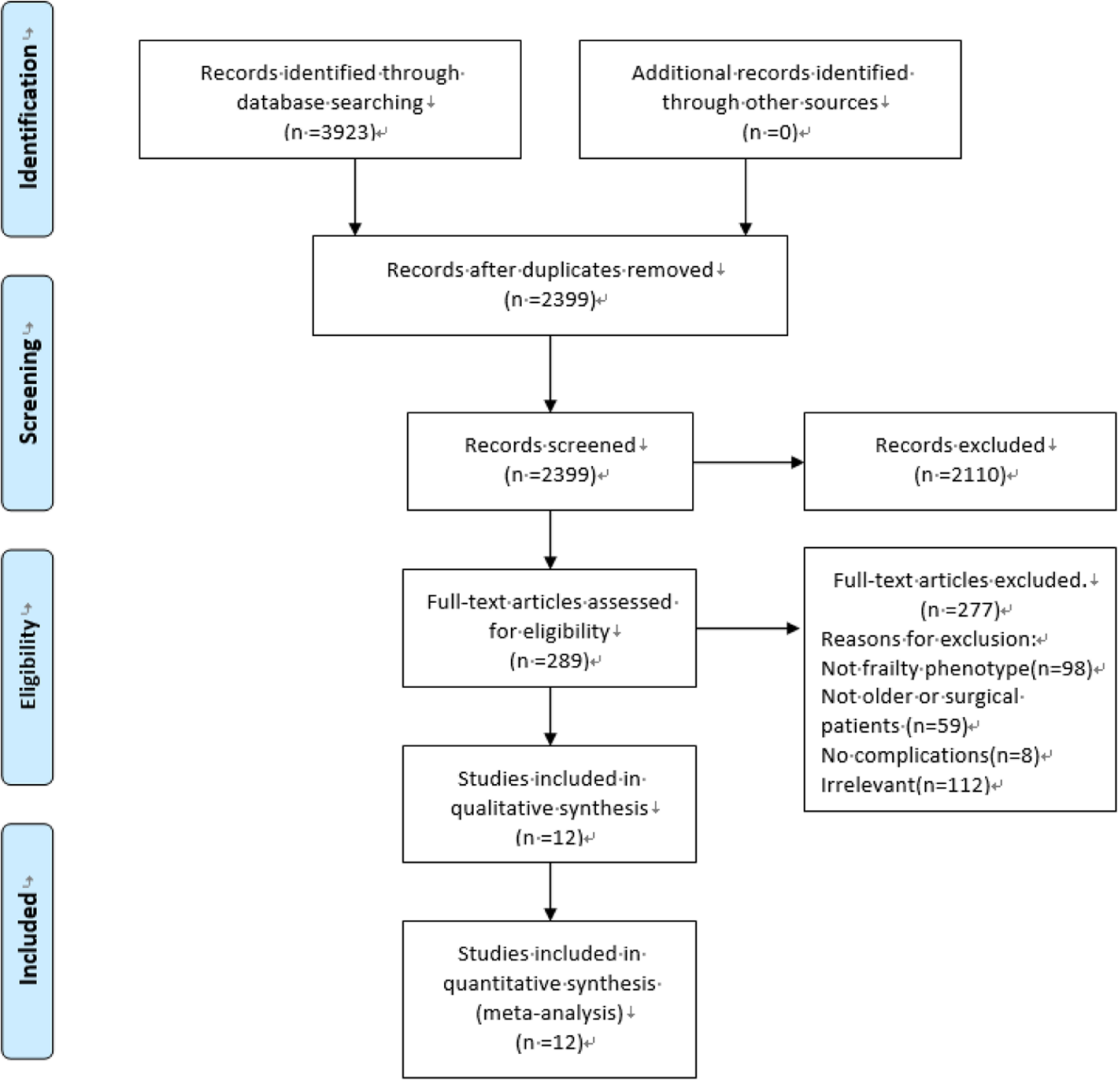
PRISMA 2009 Flow diagram of articles included in the present study

The literature search identified a total of 3923 articles (1183 from PubMed, 1492 from Web of Science, 934 from Embase and 314 from the Cochrane database). Of these, 1524 were duplicates and were removed. Two researchers reviewed the titles, abstracts and the full texts to select articles which met inclusion and exclusion criteria. In the final analysis, there were 12 articles that qualified and were included in the study [14–25]. The process of selection of the publications is depicted in Fig. 1. All 12 of the included articles were prospective observational studies published between 2010 and 2016. The study samples ranged from 25 to 594 subjects. There were two articles derived from the same database but did not address the same post-operative outcome, so we deemed these as two individual studies, which yielded a total sample of 2278 patients [15, 17]. Seven studies were conducted in the USA (America); two were from Singapore, while the others reported data from Norway, Indonesia and Spain. The reported results included those from cardiac, gastrointestinal, orthopedic, general and gynecologic surgery. Most studies evaluated short term outcomes (recorded at hospital discharge or 30-day post-operative data). If the data for the study on the Newcastle-Ottawa Scale was > 6 points, the article was regarded as being of higher quality. Detailed characteristics of the 12 relevant studies are shown on Table 1.

**Table 1:** Characteristics of included studies

Author, published year	Location	Sample size	Age	Male(%)	Type of surgery	Type of complication	Follow-up	Result (events/total)	Adjustment	NOS
Makary, 2010	USA	594	≥65 years	236 (39.7)	All types	Surgical complications defined by NSQIP	Surgery after 30 days	Frailty 15/62 Pre-frailty 40/186 Robust 47/346	Adjust age, race, gender, comorbidity, operation category, ASA, Lee score, Eagle score: Frailty VS Robust OR=2.54 (1.12,5,77) Pre-frailty VS Robust OR=2.06 (1.18,3.6)	9
Singh, 2011	America	545	≥65 years	376 (69.0)	Cardiac surgery	Death, myocardial infarction	3 years	Frailty 48/117 Non-frailty 73/428	Adjust age, gender: Frailty VS Non-frailty HR=2.61 (1.52,4.5)	8
Madeleine, 2012	America	37	≥65 years	0	gynecologic oncology patients undergoing surgery	Surgical complications which defined by NSQIP	Surgery after 30 days	Frailty 4/6 Pre-frailty 1/10 Robust 5/21	N/A	6
Gharacholou, 2012	America	545	≥65 years	376 (69.0)	cardiac surgery	major cardiovascular events	Surgery after 30 days	Frailty 11/117 Pre-frailty 30/298 Robust 10/130	N/A	8
Kristjansson, 2011	Norway	176	≥70 years	75 (42.6)	General surgery	The Clavien-Dindo classification of all type complications	Surgery after 30 days	Frailty 11/22 Pre-frailty 43/84 Robust 28/70	N/A	7
Tan, 2012	Singapore	83	≥75 years	N/A	General surgery	the Clavien-Dindo classification of type II and above complications	Surgery after 30 days	Frailty 11/23 Non-frailty 11/60	Adjust age, operation type, ASA, comorbidity, BMI, albumin: Frailty VS Non-frailty OR= 4.08 (1.43,11.64)	8
Kistler, 2015	America	35	≥65 years	6 (17.1)	Orthopedic surgery	Pneumonia, cardiac events, renal insufficiency or failure, delirium	during hospital admission	Frailty 12/18 Non-frailty 5/17	N/A	6
Ad, 2016	America	166	≥65 years	125 (75.3)	cardiac surgery	STS-defined complications and death	Surgery after 30 days	Frailty 5/39 Non-frailty 11/127	Adjust age, gender, BMI, EuroSCORE II: Frailty VS Non-frailty OR = 1.15 (0.33,3,98)	8
Cooper, 2016	America	415	≥70 years	165 (39.8)	Orthopedic surgery	major and minor complications	Surgery after 30 days	Frailty 83/145 Pre-frailty 122/223 Robust 15/47	Adjust age, gender: Frailty VS Robust RR = 1.7 (1.1,2,1) Pre-frailty VS Robust OR = 1.6 (1.1,2.1)	7
Khan, 2016	Singapore	25	>65 years	17 (68.0)	non‑cardiac major surgery	Hospital acquired infection, cardiac complications and delirium	Surgery after 10 days	Frailty 2/14 Non-frailty 2/11	Adjust cerebral oxygenation: Frailty VS Non-frailty OR = 1.27 (0.21,7.65)	7
Hamonangan, 2016	Indonesia	100	>60 years	69 (69.0)	cardiac surgery	MACE (death, myocardial infarction, and re-revascularization)	Surgery after 30 days	Frailty 5/61 Non-frailty 2/39	N/A	7
Rodriguez,2015	Spain	102	≥70 years	54 (52.9)	cardiac surgery	Heart failure	1 year	Frailty 15/29 Robust 12/73	Adjust dyslipidemia, hypertension, diabetes, and minute ventilation /carbon dioxide production slope: Frailty VS Robust OR = 4.55 (1.73, 12.01)	8

*ASA* American Society of Anesthesiologists, *NSQIP* American College of Surgeons National Surgical Quality Improvement Program, *STS* The Society of Thoracic Surgeons, *MACE* Major Adverse Cardiac Events, *HR* Hazard Ratio, *OR* odds ratio

### The association between frailty status and post-operative complications

**Fig. 2:**
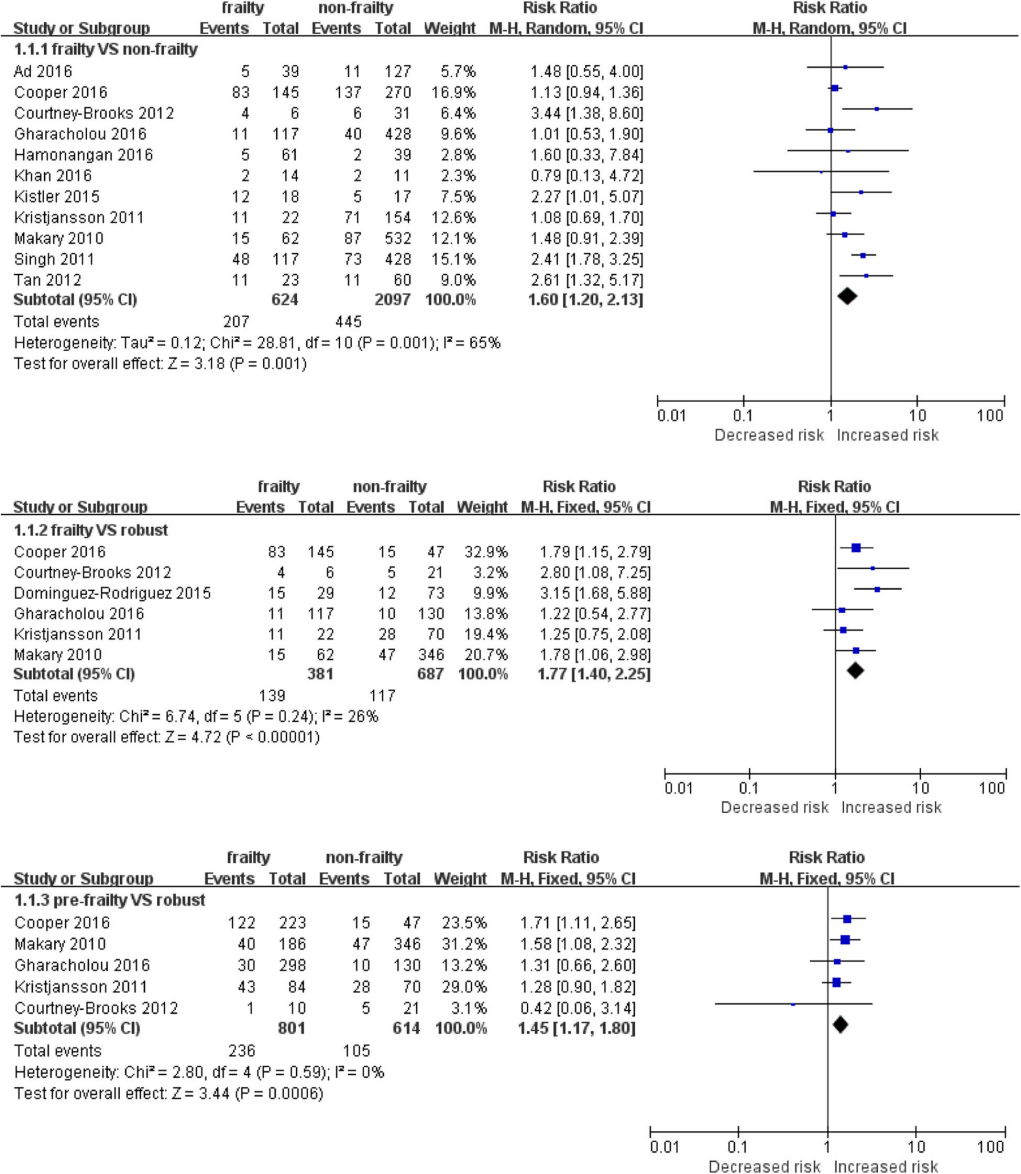
Meta-analysis of the association between frailty status and post-operative complication

To clearly illustrate the association between frailty and post-operative complications, we compared different frailty status with outcomes. We used frailty and non-frailty data from 11 publications to explore bilateral relationships, and the random-effects model was applied as a result of high heterogeneity. Figure 2 shows that the risk of post-operative complications in the frail group was higher than the non-frail group (RR: 1.6; 95% CI: 1.60–2.13). Six cohort studies reported the outcome of frail or pre-frail compared with robust patients. These results illustrated that the risk of post-operative complications in the frail group was significantly higher than the robust group (RR: 1.77; 95% CI: 1.40–2.25), and that the risk of post-operative complications in the pre-frail group was also significantly higher than that in the robust group (RR: 1.45; 95% CI: 1.17–1.80).

### Subgroup analysis

In Fig. 2, the highly heterogeneous results from the frail and non-frail groups are depicted. Subgroup analysis was performed to explore the sources of this heterogeneity. As presented in Table 2, this subgroup analysis was stratified by location, type of surgery, sample size, type of complications, follow-up period and risk adjustments. The association between outcome and frailty status was significant for the Asian studies, for studies with smaller sample sizes and shorter follow-up periods. Different surgery types and complication type may have an influence on the degree of heterogeneity. However, when combined, the adjusted OR from 3 studies showed no significant relationship between frailty and post-operative outcomes.

**Table 2:** Subgroup analysis of the relationship of frailty and post-operative complications

Items	No. of studies	Risk ratio (95% CI)	Heterogeneity	
			*P* value	I^2^
Total	11	1.6 (1.20, 2.13)	0.001	65%
Location				
America	7	1.65 (1.15, 2.38)	0.0005	75%
Asia	3	2.14 (1.18, 3.87)	0.43	0%
Type of surgery				
cardiac surgery	4	1.64 (0.96, 2.81)	0.08	55%
Non-cardiac surgery	6	1.59 (1.07, 2.37)	0.02	62%
Study sample				
<100	4	2.48 (1.60, 3.85)	0.53	0%
≥100	7	1.39 (1.01, 1.91)	0.003	70%
Type of complication				
Cardiac events	4	1.64 (0.96, 2.81)	0.08	55%
Defined by NSQIP	2	2.06 (0.91, 4.69)	0.10	62%
Defined by Clavien-Dindo	2	1.62 (0.68, 3.83)	0.03	78%
Follow-up period				
In-hospital or 30 days after surgery	8	1.59 (1.18, 2.15)	0.17	32%
≥1 year result	1	2.41 (1.78, 3.25)	N/A	N/A
Statistical analysis				
Adjusted data	3	0.77 (0.04, 1.51)	0.255	26.8%
Unadjusted data	7	1.22 (1.05, 1.43)	0.16	35%

### Sensitivity analysis and publication bias

**Fig. 3:**
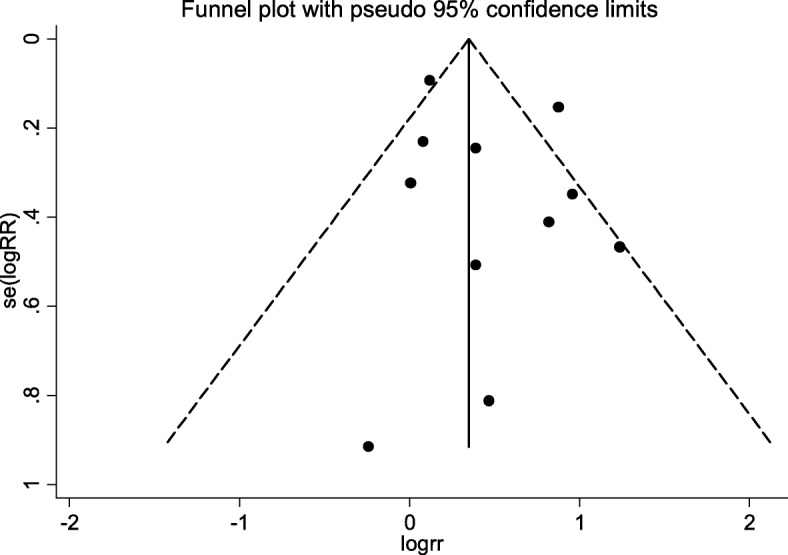
Funnel plot

When selected articles were individually removed and the effect size recalculated, the sensitivity analysis illustrated that there was no significant change of the results. In the group comparison of frailty vs. non-frailty, the effect size ranged from 1.27 (95% CI: 1.10–1.48) to 1.73 (95% CI: 1.44–2.09). Among frail or pre-frail and robust groups, the result ranged from 1.62 (95% CI: 1.25–2.10) to 1.9 (95% CI: 1.45–2.48), and 1.37 (95% CI: 1.08–1.76) to 1.53 (95% CI: 1.17–1.98), respectively. Publication bias was evaluated only for results comparing frail and non-frail groups, because the other subgroups included fewer articles and sample sizes and were not suitable for this analysis. As shown in the funnel plot (Fig. 3), no obvious publication bias was detected in this series of studies (Egger’s test, *P* = 0.755; Begg’s test, *P* = 0.343).

## Discussion

In this systematic review, 12 prospective cohort studies included 2278 older study subjects. The prevalence of frailty and pre-frailty was about 23.53 and 44.82%, respectively. Currently, most studies focus on the prevalence of frailty among community-dwelling elderly people. In the study of Collard et al. [26], the overall weighted prevalence of frailty was 10.7%, which was lower than that of the overall population of elderly patients included in this meta-analysis. Hospitalized elderly people affected by serious diseases may well have decreased organ reserves and lessened ability to respond to acute stressors, and possible worsened muscle strength and tolerance. In addition, geriatric patients are prone to nutritional deficiencies and malnutrition associated with aging, dietary restrictions, and comorbidities [27]. The frailty phenotype mainly considers the level of physical functioning of geriatric individuals, and it reflects the person’s nutritional status and exercise tolerance. Therefore, the hospitalized elderly people enrolled in this study may well have had a higher incidence and prevalence of frailty than a community-dwelling population of comparable age.

The results of the present study indicate that frailty defined by Fried’s frailty phenotype was associated with higher risk of post-operative complications in surgical patients aged 60 years and over. This is in agreement with other reviews of frailty in surgical patients. Lin et al. [28] found frailty was significantly associated with increased mortality, post-operative complications, prolonged length of stay, and discharge to residential care facility. The hypothesis that frailty predicts negative outcomes in elderly patients undergoing cardiac and gastrointestinal procedures was validated in some reviews as well [29, 30]. Surgery is a significantly stressful event that may induce a deteriorated physical status and depressed mood in the aged patient. After surgery, there may be depletion of systemic reserves, and disequilibrium of homeostasis such that health status may significantly decline in frail elderly people [31]. Moreover, the frailty phenotype entails five physical components, and single component has been used to predict adverse outcomes in past studies Chung et al. measured only the handgrip strength in 72 patients with heart failure before cardiac surgery and the results showed that the patient with grip strength lower than 25% of the body weight had a significant increase in postoperative mortality, a higher incidence of postoperative complications, and a lower survival rate [32]. Chandoo et al. reported that walking speed was associated with post-operative medical complications in elderly gastric cancer patients undergoing gastrectomy [33]. In the present subgroup analysis, the association between outcome and frailty status in Asian people may indicate that these criteria and the cut-off value for the frailty phenotype that was employed may be suitable to assess frailty in other demographic groups as well. However, there were small samples included in the available relevant publications, with a total sample of 274, and the smallest sample was 25 in one paper. Therefore, more large sample studies should be conducted.

In addition, pre-frailty patients have elevated risks for post-operative complications compared with robust patients. Given that more and more aging patients are presenting for operative procedures, frailty assessments may become a very effective tool for peri-operative evaluation and risk assessment. Evaluation of patients for frailty syndromes is recommended in the practice guidelines of the American College of Surgeons and the American Geriatrics Society [34]. However, because the definition of frailty has not been standardized, varying assessments of a surgical patient’s level of frailty used in the studies identified here have been variable. Choosing an effective frailty assessment is a serious challenge for practicing surgeons and medical staff [35]. In clinical practice, a suitable frailty assessment should be given with (a) clear purpose; (b) a theoretical basis and established validity of the underlying constructs; and (c) a feasible and convenient protocol that allows it to be utilized as a routine tool [11]. The frailty phenotype was developed from the conceptual theory of the “cycle of frailty” which includes five physical criteria [12]. Another review has shown that biomedical and physical indicators, such as nutritional status and physical function, can effectively predict adverse outcomes in hospitalized older patients [36]. In addition, it is easy and time-efficient to measure these subjective and objective criteria together as the frailty phenotype. Based on our analysis of the current literature, although 7 out of 12 articles didn’t show the significant relation between frailty and post-operative complications, the final results could provide evidence for a meaningful effect of frailty phenotype in the preoperative evaluation among geriatric patients. We think frailty phenotype, as a preoperative assessment, could efficiently identify individuals with a high risk of adverse outcomes after surgery based on the evidence of current available literature. However, the evaluation techniques applied in the 12 studies differed slightly. For instance, the cut-off point index differed from the norm in Makary’s study [14], and the definition of slowness was different in Kristjansson’s study [18]. Therefore, it is important to use a consistent definition of the frailty phenotype to enable more accurate comparisons and meta-analyses in future studies.

The strength of this review is that it includes most types of surgery (cardiac, gastrointestinal, and orthopedic) that are common in general hospitals. Compared to other studies including less types of common surgery in general hospitals, our work demonstrated that the frailty phenotype was an effective tool for surgical risk evaluation. This review provides insight into using frailty phenotypes as an effective tool for surgical risk evaluation. To our knowledge, this is the first systematic review to analyze post-operative complications based on phenotypes of physical frailty in geriatric surgical patients. However, there are some limitations in our study. Only two studies included in the analysis considered confounding factors in the design or statistics, and five articles didn’t report the influence of any confounding factors. Therefore, after we extracted the crude data from every article and calculated the summary effect, it established a causal association, and thus the strength of our results may be weak. In addition, although these articles that were included according to whether they used the frailty phenotype for defining frailty, the methods of evaluation applied in studies differed slightly. Moreover, only publications in English-language journals were included in our search, and it is possible that some relevant articles in other languages have been excluded. Previous studies indicate that frailty is a reversible process, in that exercise rehabilitation, improved nutritional support, and drug therapies may be used to modify and improve frailty status [3, 31]. Therefore, if we could screen weakened elderly patients by an effective frailty tool, then apply interventions to stabilize frailty and reduce their resulting vulnerability, this strategy may be able to decrease the rate of post-operative complications among geriatric patients. Partridge et al. have demonstrated that for patients aged 65 years or older undergoing vascular surgery, preoperative comprehensive geriatric assessment was correlated with a shorter length of hospital stay. Those receiving evaluation and optimization possessed a lower risk of complications and were less likely to be discharged to a higher level of dependency [34]. Screening frail patients is the first step to achieve benefits for these individuals, their families, and society. Our results could serve as a reference for treating clinicians and surgeons when evaluating the risk of surgery and the subsequent preoperative decision making. Besides, there is a limitation regarding how frailty is classified in the included investigations. For instance, in the Makary [14] and Courtney -Brooks [16] studies, patients having 4 or 5 of the Fried’s criteria were classified as frail whereas other included studies defined frailty as having 3 or more criteria. They also classified those with 0 or 1 criteria as “robust”, whereas other studies those having 0 criteria classified “robust”. The definition of frailty phenotype is more applicable to patients with certain self-care ability for elective surgery, and patients with critical illness or loss of self-care ability who cannot cooperate with the completion of physical assessment may need to other assessment tools to assess their frailty. Meanwhile, the frailty phenotype has been rarely applied to nonelective/ emergency surgical settings. The effect of acuity of the surgery on surgical risk evaluated by the frailty phenotype should be carried out in future. Consequently, the definition of frailty phenotype might bring more clinical benefits to elective surgical elderly patients.

## Conclusions

In summary, 12 articles were included in this systematic review and meta-analysis, and the results show that frailty phenotype is an effective assessment tool to predict risk of post-operative complications among geriatric patients. This also highlights the importance of screening frail patients by appropriate preoperative assessments. Our results could serve as a reference for the medical staff members who perform preoperative assessments and perioperative risk management in choosing a frailty measurement as a routine tool in clinical practice. Because of the limitations in our study, more strict study designs about frailty phenotype should be developed to provide stronger evidence in the future.

## Abbreviations

ASA: American Society of Anesthesiologists
CI: confidence intervals
HR: Hazard Ratio
MACE: Major Adverse Cardiac Events
NOS: The Newcastle-Ottawa Scale
NSQIP: American College of Surgeons National Surgical Quality Improvement Program
OR: odds ratio
RR: risk ratio
STS: The Society of Thoracic Surgeons
